# Pharmacokinetic and pharmacodynamic actions of clozapine-N-oxide, clozapine, and compound 21 in DREADD-based chemogenetics in mice

**DOI:** 10.1038/s41598-019-41088-2

**Published:** 2019-03-14

**Authors:** Martin Jendryka, Monika Palchaudhuri, Daniel Ursu, Bastiaan van der Veen, Birgit Liss, Dennis Kätzel, Wiebke Nissen, Anton Pekcec

**Affiliations:** 1 Boehringer Ingelheim Pharma GmbH & Co. KG, Div. Research Germany, Birkendorfer Strasse 65, 88397 Biberach an der Riss, Germany; 2 Institute of Applied Physiology, University of Ulm, Albert-Einstein-Allee 11, 89081 Ulm, Germany

## Abstract

Muscarinic Designer Receptors Exclusively Activated by Designer Drugs (DREADD) gated by clozapine-N-oxide (CNO) allow selective G-protein cascade activation in genetically specified cell-types *in vivo*. Here we compare the pharmacokinetics, off-target effects and efficacy of CNO, clozapine (CLZ) and compound 21 (Cmpd-21) at the inhibitory DREADD human Gi-coupled M4 muscarinic receptor (hM4Di). The half maximal effective concentration (EC_50_) of CLZ was substantially lower (0.42 nM) than CNO (8.1 nM); Cmpd-21 was intermediate (2.95 nM). CNO was back-converted to CLZ in mice, and CLZ accumulated in brain tissue. However, CNO itself also entered the brain, and free cerebrospinal fluid (CSF) levels were within the range to activate hM4Di directly, while free (CSF) CLZ levels remained below the detection limit. Furthermore, directly injected CLZ was strongly converted to its pharmacologically active metabolite, norclozapine. Cmpd-21 showed a superior brain penetration and long-lasting presence. Although we identified a wide range of CNO and Cmpd-21 off-targets, there was hardly any nonspecific behavioural effects among the parameters assessed by the 5-choice-serial-reaction-time task. Our results suggest that CNO (3–5 mg/kg) and Cmpd-21 (0.4–1 mg/kg) are suitable DREADD agonists, effective at latest 15 min after intraperitoneal application, but both require between-subject controls for unspecific effects.

## Introduction

Designer Receptors Exclusively Activated by Designer Drugs (DREADD) enable the direct modulation of cellular activity by activation of the Gi-, Gq- or Gs-protein-coupled signalling pathways. DREADDs have therefore emerged as a frequently used tool to dissect the neural underpinnings of behavioural functions^1^. The most widely applied DREADDs were derived from different types of muscarinic receptors and have been engineered to lose their affinity for acetylcholine, but to gain responsiveness to clozapine-N-oxide (CNO), a metabolite of the atypical antipsychotic clozapine (CLZ)^2,3^.

The value proposition and functionality of such DREADDs rests upon the assumption that their synthetic agonist is pharmacologically inert and has a reasonably high *in vivo* bioavailability in brain tissue after systemic application, two criteria that were often not met by the non-muscarinic predecessors of current DREADDs^4,5^. Recent data have questioned whether CNO does indeed fulfil these two criteria, as originally suggested^3,6^. Firstly, it has been reported that CNO can bind to alternative targets at concentrations required for DREADD activation^7^. Secondly, a study in rats raised concerns of the ability of CNO to penetrate into the brain^7^. It has been suggested that systemically applied CNO is instead converted into CLZ, and it is this CLZ, rather than CNO, that acts as the DREADD activator in brain tissue due to its strong potency at the two DREADDs, human Gq-coupled M3 muscarinic receptor (hM3Dq) and human Gi-coupled M4 muscarinic receptor (hM4Di), and its substantially higher blood-brain barrier permeability^7^. Enzymatic and non-enzymatic reduction of CNO to its corresponding base, CLZ^8^, has been demonstrated in humans^9–11^, monkeys^12^, guinea pigs^10^, rats^7,13–15^ and, recently, in mice^15,16^. Naturally, this back-conversion of CNO to CLZ, an antagonist at a wide range of G-protein coupled receptors (GPCRs), adds another source of possible off-target effects which may confound findings from *in vivo* studies – a critical issue that has been previously identified^8^. Many chemogenetic studies have not detected unspecific effects of CNO at the behavioural level in their control experiments in non-DREADD-transfected rodents^2,17^. However, a recent publication demonstrated elegantly that mice trained to report their interoceptive sensation produced by 1.25 mg/kg CLZ report such sensations after being injected with 10 mg/kg (but not 5 mg/kg or lower doses) of CNO^15^. Moreover, this back-conversion introduces difficulties in the rational design of *in vivo* studies, given that drug conversion is a factor that may vary between species, strains and sex, and depends on many additional external and intrinsic factors including metabolic capacity of the liver or health status.

To circumvent this issue, some laboratories have started using CLZ as a DREADD agonist directly, instead of CNO, at doses that do not usually cause any detectable behavioural effects in untransfected rodents. Alternatively, the recently introduced DREADD ligand, compound 21 (Cmpd-21), which is more potent than CNO at the excitatory DREADD hM3Dq, may be used^18^. A recent assessment of Cmpd-21 brain availability, behavioural off-target effects and potency at various DREADDs yielded encouraging results^16^.

However, avoiding the use of CNO as a DREADD ligand completely appears somewhat premature, especially given the dozens of successful neuroscience studies using CNO. For example, it has not yet been demonstrated that CNO itself does not enter the brain in mice. Furthermore, it has not been shown that the back-converted CLZ entering the brain is freely available to activate the DREADD (not unspecifically bound), a finding which could be more accurately measured using CNO concentration in cerebral spinal fluid (CSF) rather than in total brain tissue, as done previously^7^.

In order to enable a rational choice between the three alternative DREADD ligands (CNO, CLZ and Cmpd-21) for chemogenetic experiments in mice, we here present a large-scale pharmacokinetic, pharmacodynamic and behavioural comparison of these agents. Specifically, we directly compare efficacy at hM4Di *in vitro*, and screen for off-target binding of CNO at a large number of endogenous GPCRs. We also assess the degree and time-course of forward- and back-conversion between CNO and CLZ and measure the bioavailability of all three compounds in the brain and, importantly, CSF at multiple time points post injection. Finally, we assess unspecific effects in the 5-choice-serial-reation-time task, a test that measures a wide variety of behavioural functions.

## Results

### *In vitro* potency of CNO, CLZ and Cmpd-21 at the hM4Di receptor

**Figure 1:**
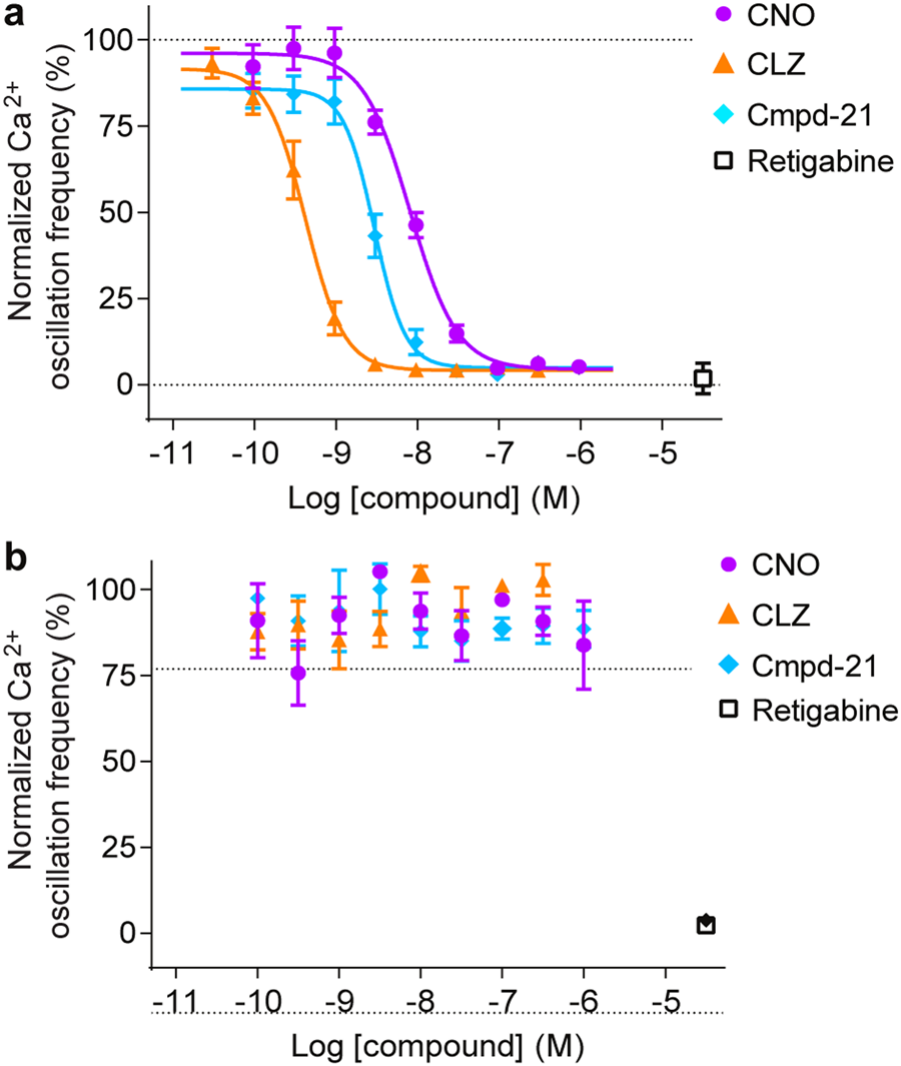
*In vitro* potency of CNO, CLZ and Cmpd-21 at hM4Di on primary embryonic rat neuronal cultures. (**a**,**b)** FLIPR assay data for measuring Ca^2+^ oscillations in (**a**) Fluo4-loaded primary rat neuronal cultures which were transduced with hM4Di-mCherry-AVV or (**b**) in control untransduced cultures. In separate experiments, CNO (purple), CLZ (orange) or Cmpd-21 (blue) were added at the indicated concentrations. The frequency of Ca^2+^ oscillations was normalised to data recorded in control wells where compounds were replaced with assay buffer. The potassium-channel opener retigabine (30 μM) was applied in control wells and served as positive control for blocking Ca^2+^ oscillations. Error bars indicate s.e.m. Each compound dose was repeated on three individual plates in duplicates. CLZ, clozapine; CNO, clozapine-N-oxide; Cmpd-21, compound 21; FLIPR, fluorescence imaging plate reader; s.e.m., standard error of the mean.

A comparative assessment of the activities of CNO, CLZ and Cmpd-21 at the inhibitory DREADD hM4Di has not previously been conducted, despite the wide usage of hM4Di for silencing of neurons and/or synapses in behaving animals. Therefore, we first conducted live calcium flux measurements via a fluorescent imaging plate reader (FLIPR) assay to evaluate the indirect activity of the above three compounds on spontaneous network activity in rat primary cultured neurons transduced with hM4Di. The potency of each agonist was measured by assessing the effect on reducing the spontaneous phasic increase in intracellular calcium (Ca^2+^ oscillations) caused by synchronous electrical activity^19^. By fitting the resulting dose-frequency curves with the Hill function (Fig. 1a), we obtained half maximal effective concentration (EC_50_)-values of 8.1 nM, 2.95 nM and 0.42 nM for CNO, Cmpd-21 and CLZ, respectively. This revealed that CNO was almost 20 times less potent than CLZ and approximately 2.7 times less potent than Cmpd-21 at hM4Di. These data are similar to EC_50_ values derived previously for hM3Dq in a FLIPR assay for CNO (6.0 nM) and Cmpd-21 (1.7 nM), but not for CLZ (1.1 nM)^18^. Application of any of the DREADD agonists alone (i.e. if neurons had not been transfected by hM4DI) had no effect on Ca^2+^oscillations in the FLIPR assay (Fig. 1b).

### Pharmacokinetic profile of CNO and CLZ after CNO application in mice

Recently, it was questioned whether CNO can efficiently penetrate the central nervous system (CNS) to activate DREADDs in rats^7^, but this has never been examined in mice. Therefore, we systematically estimated free drug levels of CNO and CLZ in the CNS from concentrations in CSF, in addition to measuring their concentrations in cortical brain tissue and blood plasma.

**Figure 2:**
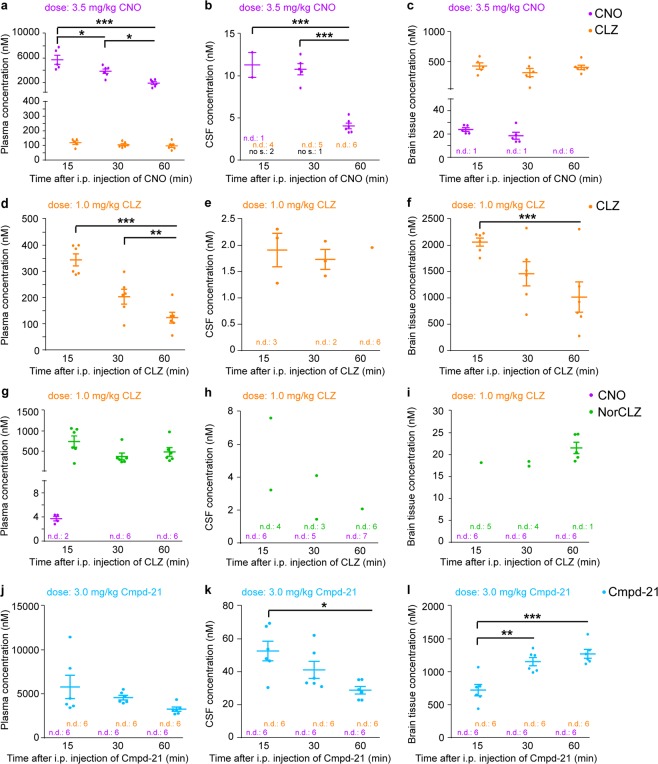
*In vivo* pharmacokinetic profile of CNO, CLZ and Cmpd-21. (**a–c)**: Concentration (nM) of CNO (purple) and CLZ (orange) at 15, 30 and 60 min after i.p. injection of 3.5 mg/kg CNO measured in (**a**) plasma, (**b**) CSF, and (**c**) cortical brain tissue. (**d–f**): Concentration (nM) of CLZ (orange) at 15, 30 and 60 min after i.p. injection of 1.0 mg/kg CLZ in (**d**) plasma, (**e**) CSF, and (**f**) cortical brain tissue. (**g–i**): Concentration (nM) of CNO (purple) and NorCLZ (green) at 15, 30 and 60 min after i.p. injection of 1.0 mg/kg CLZ in (**g**) plasma, (**h**) CSF, and (**i**) cortical brain tissue. (**j–l**): Concentration (nM) of Cmpd-21 (blue) at 15, 30 and 60 min after i.p. injection of 3.0 mg/kg Cmpd-21 in (**j**) plasma, (**k**) CSF, and (**l**) cortical brain tissue. Mostly, for each drug and time point n = 6 animals were used and analysed, except for the 15 min time-point after CNO application (n = 5) and the CSF samples after CLZ injection (**e**,**h**) where 5 samples were taken at 30 min and 7 samples at 60 min. Each sample is displayed as an individual dot in the respective graph. Concentrations of CNO (purple) and CLZ (orange) were measured in all (**a–l**) samples, but were not detected (n.d.) in the number of samples stated in the respective panel for the respective compound (CNO: purple font; CLZ: orange font; NorCLZ: green font, only measured after CLZ injection), as either no clear peak was identified or the concentration remained below the detection limit. The detection limit for CNO and CLZ was 1–2.5 nM depending on sample quality, and for NorCLZ was 15 nM. Additionally, CSF sampling failed in 3 animals (no s., black font), as indicated in (**b**). Significant differences represent pair-wise Tukey *post hoc* tests conducted after a significant result in the one-way ANOVA across all time points. *p < 0.05, **p < 0.01, ***p < 0.001. Horizontal bars represent mean (wide) and s.e.m. (narrow); they are omitted if the respective substance remained undetectable in >50% of samples. ANOVA, analysis of variance; CLZ, clozapine; CNO, clozapine-N-oxide; Cmpd-21, compound 21; CSF, cerebrospinal fluid; i.p., intraperitoneal; NorCLZ, norclozapine; s.e.m., standard error of the mean.

We found that, in mice, CNO does enter the brain. After systemic administration of 3.5 mg/kg CNO, free CNO levels (in CSF) and total brain concentration were both higher than the EC_50_ at hM4Di and hM3Dq (see above) from at least 15 min post injection until at least 30 min post injection (Fig. 2b). By 60 min post injection, however, free CNO levels had dropped surprisingly sharply, to about half the EC_50_ at hM4Di, 4.05 nM. In cortex tissue, CNO could not be detected at the final time point (Fig. 2c).

We also confirmed the recent observation that CNO is back-converted to CLZ in mice^15,16^. Such back-conversion is known to occur in humans and is likely of hepatic nature (i.e. CYP enzyme-mediated)^9,10^. In mice, this back-conversion appears to occur quickly. Mean CLZ concentrations, as with mean CNO concentrations, reached maximum plasma concentrations (C_max_) at the first sampling time point, 15 min post injection (Fig. 2a).

Our data also confirmed that CLZ penetrates the brain to a greater extent than CNO^7^, appearing to accumulate in the brain tissue. While the mean CLZ plasma concentration corresponded to approximately 3.3% of the mean CNO plasma concentration 15 min post 3.5 mg/kg CNO injection, the mean brain CLZ concentration was approximately 20 times the mean brain CNO concentration at this time point (Fig. 2c). Furthermore, the absolute CLZ levels in brain after injection of 3.5 mg/kg CNO, approximately 427 nM, or more than 1000 times the EC_50_ at hM4Di, were barely diminished at the 60-min time point (mean, 406 nM).

At all sampling points, CLZ concentration in CSF remained below the detection limit of 1–2.5 nM. However, since this is in the range of the EC_50_ at both hM4Di and hM3Dq^18^, we cannot rule out that there would still be enough free CLZ to activate both DREADDs efficiently. Nevertheless, we can conclude that the very high levels of CLZ in the brain do not translate proportionally into CSF levels, while the comparatively lower brain levels of CNO do (mean CNO concentration in brain: 23.8 and 18.5 nM at 15 and 30 min, respectively; mean CNO concentration in CSF: 11.2 and 10.8 nM at 15 and 30 min, respectively; Fig. 2b,c).

### Pharmacokinetic profile of CLZ after direct application in mice

To determine the suitable dose range for application of CLZ as a DREADD agonist, we repeated the *in vivo* pharmacokinetic assessment presented above after intraperitoneal (i.p.) injection of 1 mg/kg CLZ. As seen with CNO, levels of CLZ peaked at the first point of measurement, 15 min post injection, in plasma (344 nM), brain (2063 nM) and CSF (1.9 nM, Fig. 2d–f). While CSF concentrations remained largely constant across all time points of sampling, plasma and brain levels decreased linearly across those time points reaching 123 nM (plasma) and 1016 nM (brain), respectively, at 60 min (Fig. 2d,f). The absolute concentrations and their kinetics were in line with an accumulation and potentially unspecific binding of CLZ in the brain, as seen after CNO injection.

Additionally, we found a small degree of conversion of CLZ to CNO, exclusively at the first time point (15 min) in plasma, where the mean CNO concentration reached approximately 1.1% of the CLZ concentration in plasma (Fig. 2g). At all other time points and in the other sample types, no CNO was detectable (Fig. 2g–i). In contrast, the alternative CLZ metabolite norclozapine (NorCLZ) was detectable in all plasma samples, a minority of CSF samples, and, especially at later time points, in brain tissue samples (Fig. 2g–i). Given that the mean brain concentration of CLZ was nearly 6 times higher than the plasma concentration at the first time point, the absence of CNO in brain tissue suggests a lack of CLZ-to-CNO forward-conversion in the CNS.

### Pharmacokinetic profile of Cmpd-21 in mice

To complete the comparative analysis, the pharmacokinetic assessment was repeated using 3.0 mg/kg Cmpd-21. Plasma and CSF levels peaked at the earliest time point of measurement, 15 min post injection, with mean concentrations of 5833 nM and 52 nM, respectively (Fig. 2j,k). The plasma and CSF concentrations decreased gradually reaching 57% and 54% of peak values at 60 min post injection, respectively. Mean brain concentrations, in turn, increased over time from 722 nM (15 min post injection) to 1273 nM (60 min post injection; Fig. 2l). This demonstrates that Cmpd-21 re-distributes in the body and enriches in the CNS compartment. Finally, we did not detect any conversion of Cmpd-21 to CLZ or CNO in mice (data not shown).

### Off-target effects of CNO

Due to the comparatively high EC_50_ at DREADDs and low bioavailability in the brain, the use of CNO requires relatively high doses, which in turn poses the risk of off-target effects on other receptors. While the first publication introducing DREADDs suggested a lack of significant off-target effects of nanomolar concentrations of CNO^3^, a more recent investigation concluded that 10 μM CNO may competitively inhibit binding of ligands to a range of endogenous GPCRs^7^. However, according to our data, this concentration is more than 10 times the brain concentrations and 1000 times the CSF concentrations that are reached after injection of 3.5 mg/kg CNO in mice.

**Figure 3:**
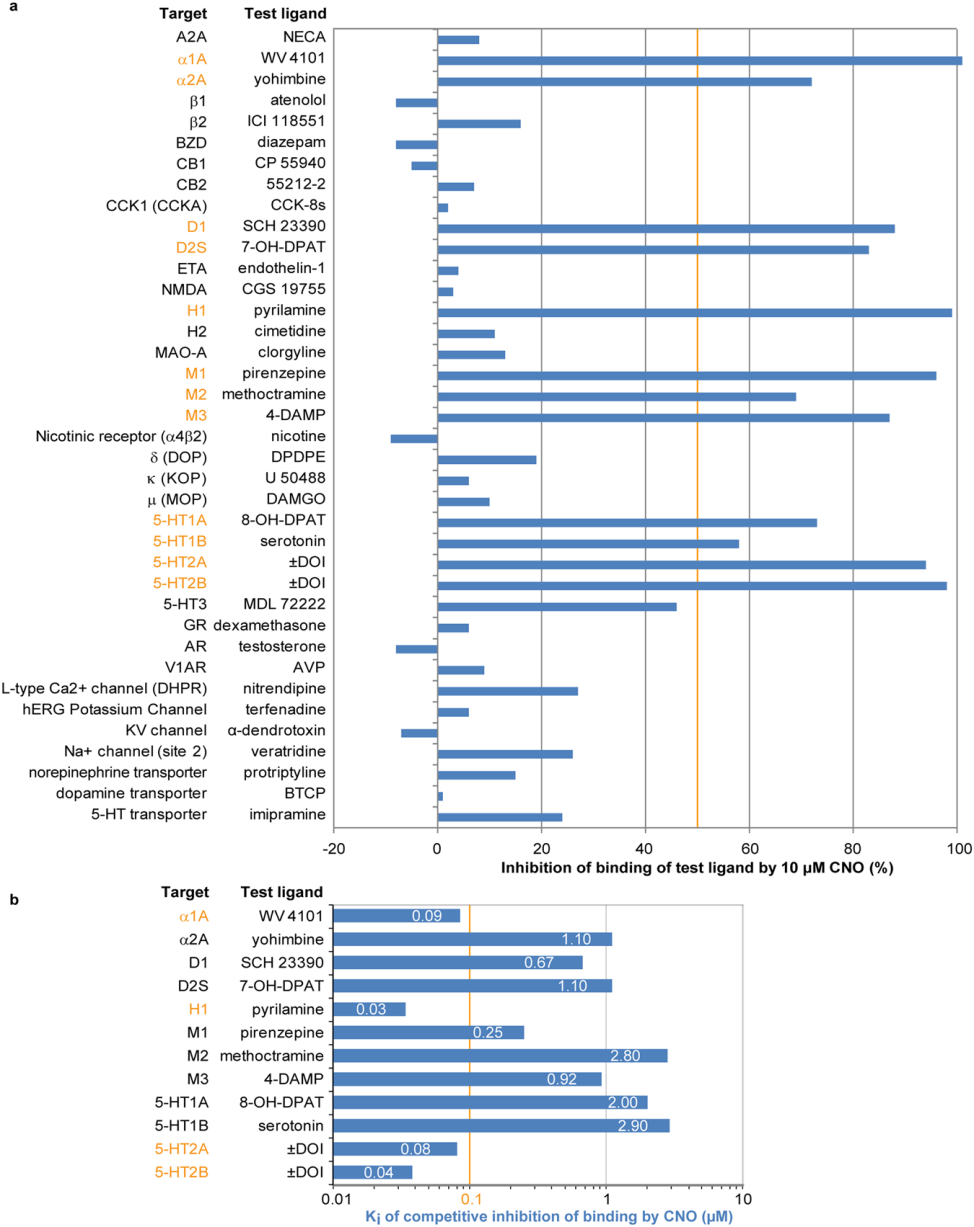
Off-target screen for CNO. (**a**) Radioligand assay determining the % inhibition of binding of the indicated test ligand to the stated target caused by 10 μM CNO. Results showing an inhibition >50% (crossing the orange vertical line) are considered to represent significant effects of CNO, and thereby highlight potential off-target effects (orange font). Note that 6 enzymes, COX1, COX2, PDE3A, PDE4O2, Lck kinase, and AChE, were probed in the same assay, but all resulted in an inhibition <20% (not shown). All values are the mean of 2 replicates of the assay. (**b**) K_i_ of CNO at the 8 significant off-target receptors identified in screen (**a**), as derived from concentration binding assays and calculated using the Cheng-Prusoff equation. Orange font indicates targets with a K_i_ < 100 nM. Values have been derived by non-linear regression of the competition curves generated with mean values of 2 replicates at 8 different concentrations of CNO (3*10^−10^–1*10^−4^ M) using Hill equation curve fitting. 4-DAMP, 1,1-dimethyl-4-diphenylacetoxypiperidinium iodide; 5-HT, serotonin; A2A, adenosine; AChE, acetylcholinesterase; AR, androgen receptor; AVP, arginine vasopressin; BTCP, 1-[1-(2-Benzo[b]thiopheneyl)cyclohexyl]piperidine hydrochloride; BZD, benzodiazepine; CB, cannabinoid receptor; CCK, cholecystokinin; CGS 19755, selfotel; CNO, clozapine-N-oxide; COX, cyclooxygenase; D, dopamine; DAMGO, [D-Ala^2^, N-MePhe^4^, Gly-ol]-enkephalin; DPDPE, [D-Pen^2^, D-Pen^5^]enkephalin; DOI, 2,5-Dimethoxy-4-iodoamphetamine; ETA, endothelin receptor; GR, glucocorticoid receptor; H, histamine; hERG, human ether-a-go-go-related gene; K_i_, inhibition constant; KV, potassium channel; MAO-A, monoamine oxidase A; M, muscarinic; MDL 72222, bemesetron; NMDA, *N*-methyl-D-aspartate; NECA, N-Ethyl-5′-carboxamido adenosine; PDE, phosphodiesterase; SCH 23390, halobenzazepine; V1A, vasopressin receptor 1 A.

We therefore comprehensively evaluated the potential for off-target effects following CNO administration in our experimental model. Firstly, we re-assessed the potential of 10 μM CNO to bind to 44 potential alternative targets (Fig. 3a), confirming previously published targets and identifying further GPCRs at which CNO showed over 50% competitive inhibition of ligand binding. These included α1 A and α2 A adrenoreceptors, H1 histamine receptor, M1, M2 and M3 muscarinic receptors, 5-HT1A, 5-HT1B, 5-HT2A and 5-HT2B serotonin receptors, D1 and D2 dopamine receptors (Fig. 3a). We then identified the inhibitory constant (K_i_) for CNO at the 12 identified GPCRs. Particularly high, namely sub-μM, affinities of CNO were found at α1A, H1, M1, 5-HT2A and 5-HT2B receptors (Fig. 3b).

Additionally, we re-assessed the potency of CNO and CLZ at the parent receptor of hM4Di, the human M4 muscarinic receptor, and found EC_50_ values of 6300 nM and 21 nM, respectively (data not shown). These correspond to 777 times and 52 times the EC_50_ values at hM4Di for CNO and CLZ, respectively, leaving sufficient room for selective DREADD activation by both compounds.

### Off-target effects of Cmpd-21

**Figure 4:**
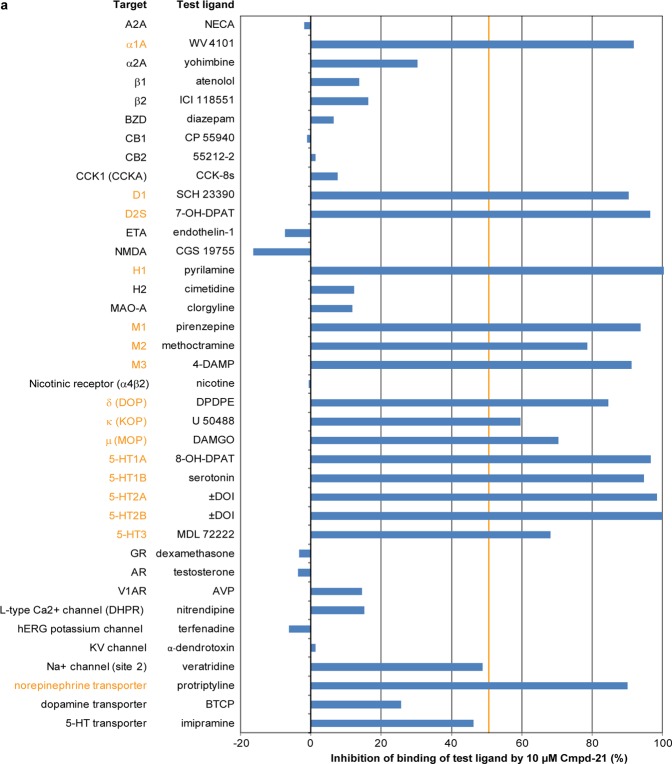
Off-target screen for Cmpd-21. (**a**) Radioligand assay determining the % inhibition of binding of the indicated test ligand to the stated target caused by 10 μM Cmpd-21. Results showing an inhibition >50% (crossing the orange vertical line) are considered to represent significant effects of Cmpd-21, and thereby highlight potential off- target effects (orange font). Note that 6 enzymes, COX1, COX2, PDE3A, PDE4D2, Lck kinase and AChE, were probed in the same assay, with all but one showing an inhibition <25%; at COX2 Cmpd-21 exerted 60% inhibition of ligand binding (not shown). All values are the mean of 2 replicates of the assay. 4-DAMP, 1,1-dimethyl-4-diphenylacetoxypiperidinium iodide; 5-HT, serotonin; A2A, adenosine; AChE, acetylcholinesterase; AR, androgen receptor; AVP, arginine vasopressin; BTCP, 1-[1-(2-Benzo[b]thiopheneyl)cyclohexyl]piperidine hydrochloride; BZD, benzodiazepine; CB, cannabinoid receptor; CCK, cholecystokinin; CGS 19755, selfotel; CNO, clozapine-N-oxide; COX, cyclooxygenase; D, dopamine; DAMGO, [D-Ala^2^, N-MePhe^4^, Gly-ol]-enkephalin; DPDPE, [D-Pen^2^, D-Pen^5^]enkephalin; DOI, 2,5-Dimethoxy-4-iodoamphetamine; ETA, endothelin receptor; GR, glucocorticoid receptor; H, histamine; hERG, human ether-a-go-go-related gene;K_i_, inhibition constant; KV, potassium channel; MAO-A, monoamine oxidase A; M, muscarinic; MDL 72222, bemesetron; NMDA, *N*-methyl-D-aspartate; NECA, N-Ethyl-5′-carboxamido adenosine; PDE, phosphodiesterase; SCH 23390, halobenzazepine; V1A, vasopressin receptor 1A.

Cmpd-21 has previously been suggested to be favourable to CNO due to the lack of back-conversion to CLZ or significant off-target effects, relative to its high potency at DREADDs^18^. A previous study, however, found evidence for competitive binding to D1, D2 and M4 receptors^16^. We therefore evaluated the binding of Cmpd-21 to a wide range of GPCR and non-GPCR targets using 10 μM Cmpd-21. Surprisingly, this dose produced a significant (>50%) competitive inhibition of binding to a wide range of endogenous GPCRs, including the α1A-adrenoreceptor, the H1 histamine receptor, and all tested dopamine (D1, D2), muscarinic (M1, M2, M3), opioid (δ, κ, μ), and serotonin (5-HT1A, 5-HT1B, 5-HT2A, 5-HT2B, 5-HT3) receptors (Fig. 4).

### Effects of CNO, CLZ and Cmpd-21 in the 5-CSRTT

Given the potential of off-target effects of all three compounds, we assessed the influence of CNO, CLZ and Cmpd-21 on a wide range of behavioural measures, including sustained attention, inattentiveness, impulsivity, locomotor activity, motivation and perseveration using the 5-choice serial-reaction-time task (5-CSRTT)^20^. On days of drug injection, a behavioural protocol was applied that challenged attention and impulsivity by decreasing the presentation time of the stimulus from 2 s to 0.8 s and increasing the waiting time before stimulus onset from 5 s to 7 s (relative to the baseline protocol).

**Figure 5:**
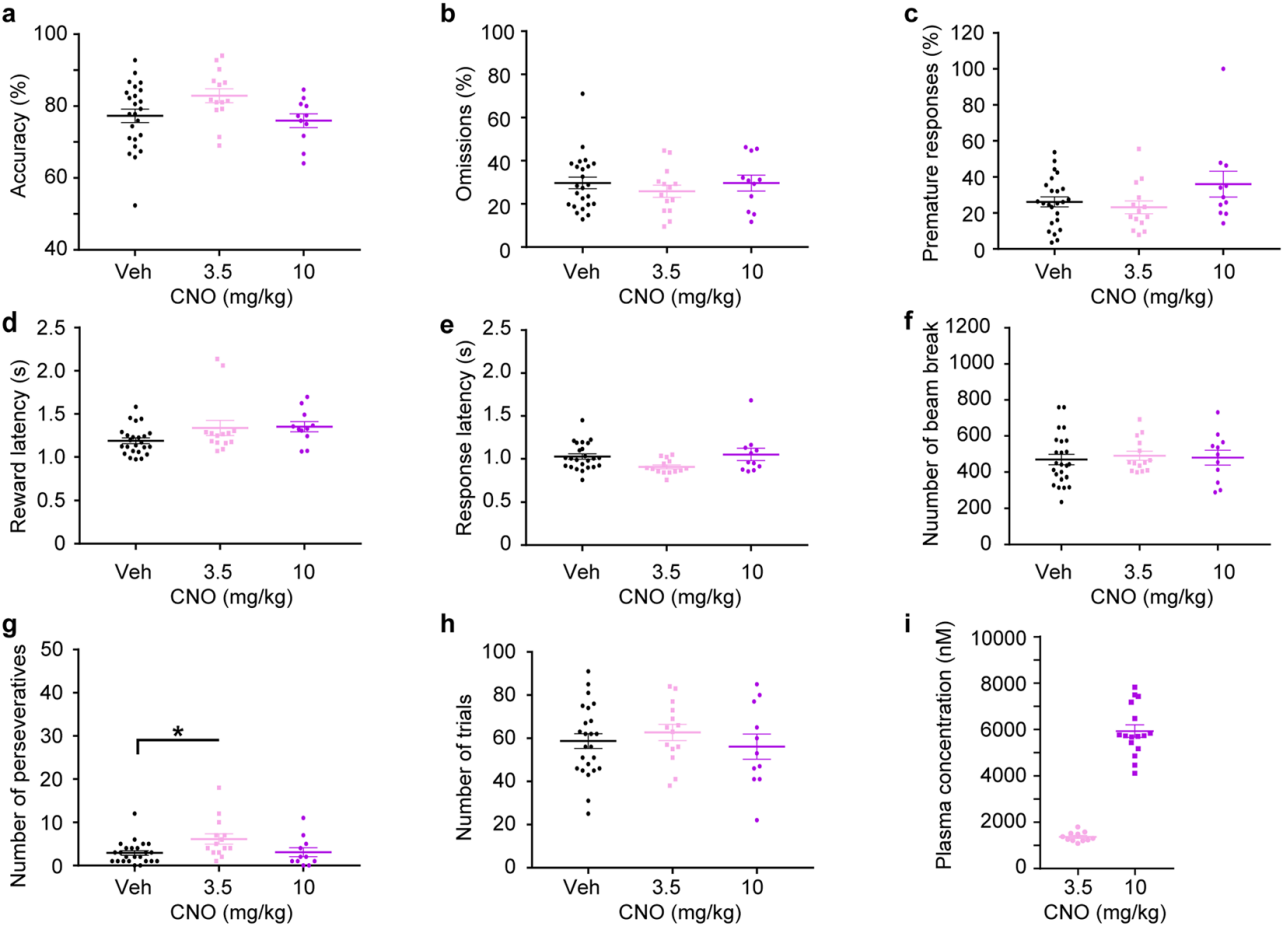
Assessment of unspecific behavioural effects of CNO in the 5-CSRTT. (**a**) Attentional accuracy (%); (**b**) relative number of omitted responses (%); (**c**) relative number of premature responses (%); (**d**) latency to collect the reward after correct responses (s); (**e**) latency to make a correct response after the onset of the stimulus (s); (**f**) number of beam breaks caused by movement between the 5-choice wall and the receptacle; (**g**) number of perseverative (repeated) responses into the same correct hole; and (**h**) total number of trials conducted by the animal within a 30-min session during the 5-CSRTT started 10 min after i.p. injection of either vehicle (Veh, black), 3.5 (pink) or 10 mg/kg CNO (purple), as indicated. (**i**) Plasma concentrations of CNO (nM), determined 45 min after i.p. injection; statistical differences not indicated. *p < 0.05, Dunnett’s *post hoc* test comparing the value under 3.5 or 10 mg/kg CNO to the vehicle value after significant one-way ANOVA across all three groups. Note that experiments using 3.5 vs 10 mg/kg were conducted separately, each following a between-subject design; data from both vehicle groups were combined. The starting dose was counter-balanced across animals, in each case. Results are shown as data from individual animals (dots) and as population mean ± s.e.m. (horizontal lines). 5-CSRTT, five-choice serial-reaction time task; ANOVA, analysis of variance; CNO, clozapine-N-oxide; i.p., intraperitoneal; s.e.m., standard error of the mean.

CNO, at doses of 3.5 or 10 mg/kg tested in two consecutive between-subject experiments, did not produce any significant behavioural changes in most 5-CSRTT parameters relative to the combined vehicle-group from both experiments. The only notable effect was a modest increase of the number of perseverative responses at the intermediate dose (*p* = 0.0142, Dunnett’s *post hoc* test; *p* = 0.0196 for one-way analysis of variance [ANOVA] across all three conditions), but not at the highest dose (Fig. 5a–h).

**Figure 6:**
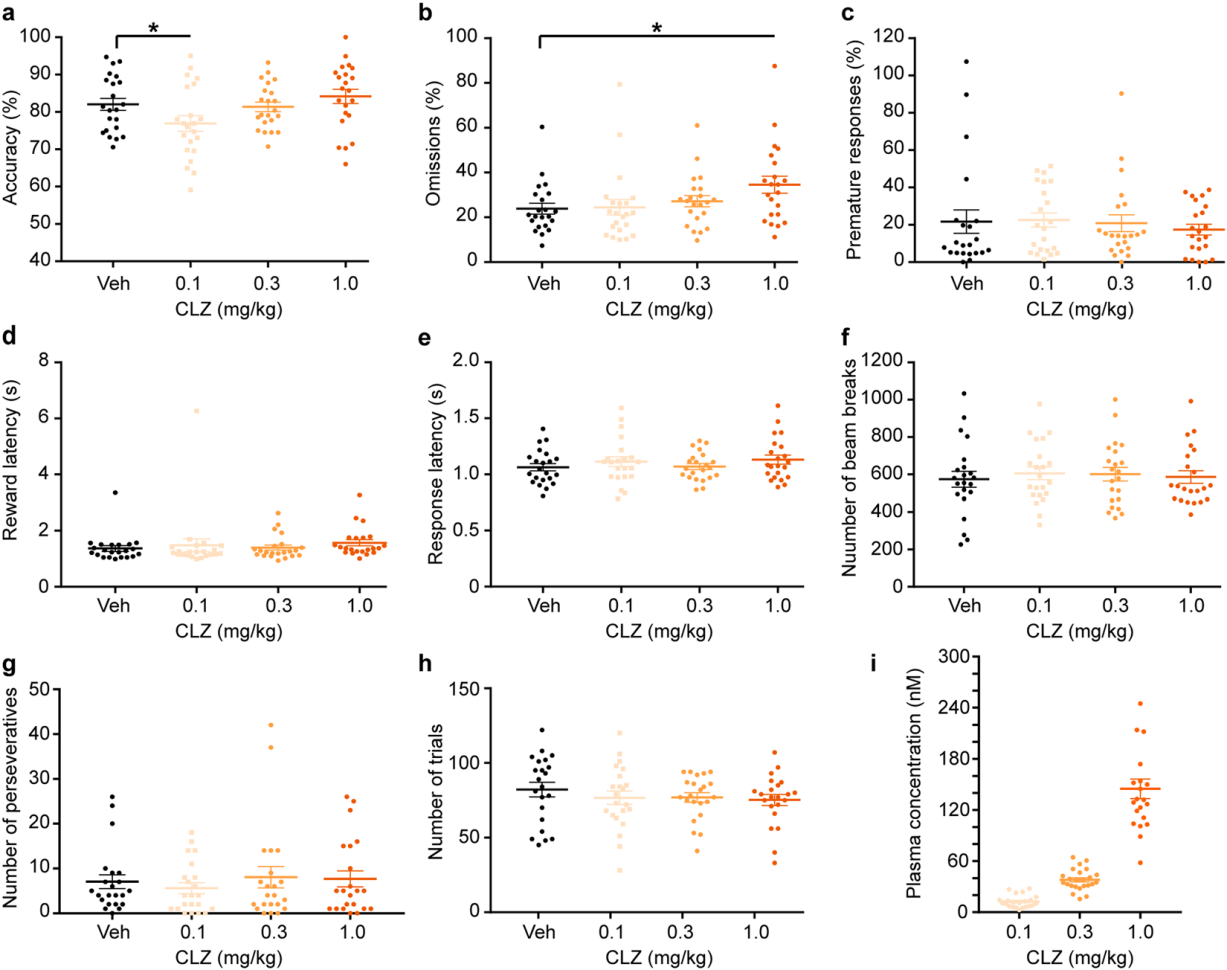
Assessment of unspecific behavioural effects of CLZ in the 5-CSRTT. (**a**) Attentional accuracy (%); (**b**) relative number of omitted responses (%); (**c**) relative number of premature responses (%); (**d**) latency to collect the reward after correct responses (s); (**e**) latency to make a correct response after the onset of the stimulus (s); (**f**) number of beam breaks caused by movement between the 5-choice wall and the receptacle; (**g**) number of perseverative (repeated) responses into the same correct hole; and (**h**) total number of trials conducted by the animal within a 30-min session during the 5-CSRTT started 10 min after i.p. injection of either vehicle (Veh, black), 0.1 (yellow), 3.5 (orange) or 10 mg/kg CLZ (red), as indicated. (**i**) Plasma concentrations (nM) of CLZ determined 45 min after i.p. injection; statistical differences not indicated. Note that all doses were tested within one within-subject experiment, following a latin-square design in which the starting dose was counter-balanced across animals. *p < 0.05; indicated are pairwise differences assessed after a significant effect of dose in a repeated-measures ANOVA using Dunnett’s *post hoc* test comparing the value under a given dose of CLZ to the vehicle value. Results are shown as data from individual animals (dots) and as population mean ± s.e.m. (horizontal lines). 5-CSRTT, five-choice serial-reaction time task; ANOVA, analysis of variance; CLZ, clozapine; i.p., intraperitoneal; s.e.m., standard error of the mean.

0.1, 0.3 and 1 mg/kg of CLZ did not produce any significant behavioural changes in most 5-CSRTT parameters, except for a mild decrease of attentional accuracy at 0.1 mg/kg (*p* = 0.0439, paired Dunnett’s *post hoc* test conducted after significant effect of dose in the repeated-measures ANOVA) and a mild increase of omissions, indicating inattentiveness, at 1 mg/kg (*p* = 0.0108, as before) compared with vehicle (Fig. 6a–h).

**Figure 7:**
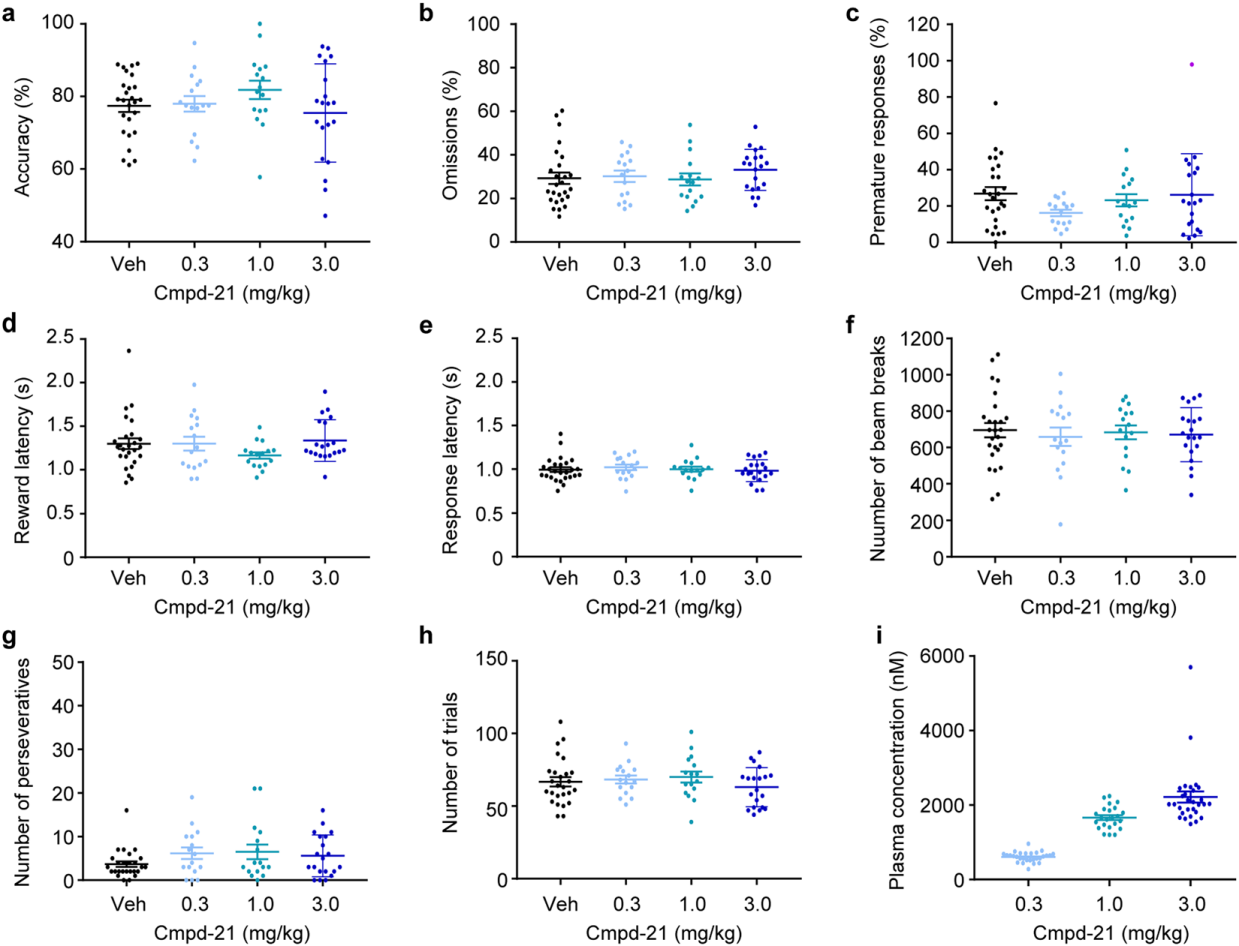
Assessment of unspecific behavioural effects of Cmpd-21 in the 5-CSRTT. (**a**) Attentional accuracy (%); (**b**) relative number of omitted responses (%); (**c**) relative number of premature responses (%); (**d**) latency to collect the reward after correct responses (s); (**e**) latency to make a correct response after the onset of the stimulus (s); (**f**) number of beam breaks caused by movement between the 5-choice wall and the receptacle; (**g**) number of perseverative (repeated) responses into the same correct hole, and (**h**) total number of trials conducted by the animal within a 30-min session during the 5-CSRTT started 10 min after i.p. injection of either vehicle (Veh, black), 0.3 (light blue), 1.0 (green) or 3.0 mg/kg compound 21 (Cmpd-21, blue), as indicated. (**i**) Plasma levels of Cmpd-21 determined 45 min after i.p. injection; statistical differences not indicated. Note that the plots contain combined data from two separate, consecutive experiments, one applying 3 mg/kg and the other applying 0.3 and 1.0 mg/kg Cmpd-21, whereby each had its own within-subject vehicle control. Hence, for statistical analysis, behavioural data is not compared between the dose levels of those separate experiments. Instead, a repeated-measures ANOVA was conducted for the experiment including 0.3 and 1.0 mg/kg, and a paired *t*-test was done to analyse the experiment including 3.0 mg/kg Cmpd-21. No significant differences were found for any measures. For both within-subject experiments the starting dose was counter-balanced across animals. Results are shown as data from individual animals (dots) and as population mean ± s.e.m. (horizontal lines). 5-CSRTT, five-choice serial-reaction time task; ANOVA, analysis of variance; Cmpd-21, compound 21; i.p., intraperitoneal; s.e.m., standard error of the mean.

0.3, 1 and 3 mg/kg of Cmpd-21 did not produce any significant behavioural changes in any 5-CSRTT parameters assessed (Fig. 7a–h).

During the 5-CSRTT task, plasma concentrations for all drugs were determined 45 min post injection (Figs 5i, 6i, 7i) and corresponded generally well with the pharmacokinetic profiles obtained separately before (Fig. 2) and with the expected dose-related concentration differences.

## Discussion

Here, we conducted the first systematic comparative assessment of the pharmacokinetics and unspecific behavioural effects of the DREADD agonists CNO, CLZ, and Cmpd-21 in mice, and related it to measurements of their efficacy at the inhibitory DREADD hM4Di.

Our data strengthens the notion that CNO is back-converted to CLZ in mice^15,16^, as recently also shown in rats^7^. However, in contrast to the conclusion from a previous study on this back-conversion in rats^7^, our data does not support the view that the gating of DREADDs expressed in the CNS results solely from back-converted CLZ after CNO application. Instead, our study shows that, in mice, unbound CNO is present in the brain at sufficient levels to activate the DREADDs directly, after injection of a 3.5 mg/kg dose. While some publications using the excitatory DREADD hMD3q report behavioural effects of CNO at doses less than 0.5 mg/kg^2^, studies using hM4Di usually apply doses between 0.5 and 10 mg/kg^2,17,20,21^ (i.e. a dose range at which CNO itself could act as the agonist in brain tissue). One explanation for this discrepancy is that, although the concentrations of back-converted CLZ in brain tissue after CNO administration are impressively high, the vast majority of this CLZ might be bound unspecifically to tissue and not be actually available for DREADD activation. This interpretation is supported by our measurement of both compounds in CSF, which approximates the proportion of free ligand levels in the brain more accurately than total brain or plasma concentrations^22^. Therefore, our data supports the hypothesis that certainly CNO, and only potentially back-converted CLZ, contribute to DREADD activation in the CNS of mice after systemic application of CNO. A DREADD engagement of both CNO and its active metabolite CLZ is specifically conceivable when using higher CNO doses (>1–2 mg/kg) administered intraperitoneally. Against the backdrop of the current literature, our data underlines that the degree of back-conversion of CNO to CLZ and the brain availability of both compounds is species-specific^7,15^.

The *in vivo* pharmacokinetic assessment of CLZ on its own, administered as an i.p. injection of 1 mg/kg, indicated an accumulation of CLZ in the brain. This fast penetration into the brain is likely because it is highly lipophilic, implying that it binds unspecifically to the tissue parenchyma, resulting in initially high brain concentrations. The observation that the free (CSF) concentration of CLZ corresponds to approximately 0.1% of the brain concentration and 1% of the plasma concentration suggests that the CLZ CSF levels after injection of 3.5 mg/kg CNO would unlikely exceed the EC_50_ of hM4Di (0.4 nM). Assuming a linear increase in the kinetic profile by dose, our data indicate that a dose range between 0.2 and 0.5 mg/kg of CLZ should be used in experiments involving hM4Di and between 0.5–1 mg/kg should be used to activate hM3Dq.

Interestingly, we also found some evidence of a forward*-*conversion of CLZ to CNO. However, the CNO doses were only detectable at 15 min post injection in plasma and were too low to hypothesize a competition for DREADD activation in the CNS. Also, given that the mean brain concentration of CLZ was nearly 6 times higher than the plasma concentration at that first time point, the absence of CNO in brain tissue suggests a lack of CLZ-to-CNO forward-conversion in the CNS. In contrast, the degree of conversion of CLZ to its alternative and pharmacologically active metabolite, NorCLZ, was substantially higher than its conversion to CNO. Notably, this differs from data using rats showing a preferential conversion of CLZ into CNO rather than NorCLZ^15^. In humans, NorCLZ and CNO are the two primary active metabolites created through hepatic metabolism, with the former being more active^23^. NorCLZ has been shown to have higher antagonist activity at 5-HT1C and 5-HT2C serotonin receptors, as well as D1 and D2 dopamine receptors, compared with the parent compound. It also has additional activity as an M1 muscarinic receptor partial agonist (reviewed in Ellison & Dufresne, 2015^24^). Considering this distinct off-target activity and considering that the pharmacokinetic profile and brain tissue penetration rate of NorCLZ differs from that of CLZ in humans^23^, it is at least conceivable that the formation of NorCLZ in mice may confound the behavioural readout. Based on these data, CNO and Cmpd-21 may be preferred as DREADD ligands in mice, at least until the potency of NorCLZ on DREADDs and its pharmacokinetic profile in mice has been fully elucidated.

Notably, the kinetics of CLZ differed depending on whether it was applied directly or originated from injected CNO. A single injection of CLZ resulted in initially high plasma and brain concentrations that decreased relatively quickly over time due to its clearance. In our experiments, the decline within 60 min in plasma and brain tissue was approximately 2-fold. However, in the case of CNO administration, CLZ plasma levels proved to be relatively stable over the course of 60 min, which in turn, was mirrored by a stable CLZ brain tissue concentration. This is likely due to the fact that the CNO-to-CLZ back-conversion happens at a constant rate over time in mice, as in humans^9^. Thereby, CNO generation and clearance should reach an equilibrium, and the supply of CLZ occurs akin to a continuous infusion rather than a single injection.

The pharmacokinetic profile of Cmpd-21 indicated that CSF levels were almost 10 times the measured EC_50_ at hM4Di and about 16 times the published EC_50_ at hM3Dq^18^ (1.7 nM) after systemic administration of a dose of 3 mg/kg. Therefore, doses between 0.4 and 1 mg/kg should be sufficient to render a strong activation of both DREADDs in mice for approximately 1 h post injection. Furthermore, we did not observe any conversion to CLZ or CNO, which makes Cmpd-21 an attractive alternative DREADD agonist.

In addition to the off-target effects potentially resulting from back-converted CLZ, CNO itself might also act on alternative endogenous receptors^7^. We found that the *K*_i_ of CNO binding reaches <100 nM at some GPCRs, including most notably the H1 histamine, the 5-HT2A and the 5-HT2B serotonin receptors. Interestingly, we also found strong competitive binding of Cmpd-21 to receptor sites of dopamine, serotonin, opioid, muscarinic, histamine and adrenoreceptors. Despite the surprisingly large range of off-targets for all three DREADD agonists, we found only very subtle, if any, behavioural alterations in our assessment using the 5-CSRTT^14,15^. This suggests that the doses used for DREADD activation are still lower than would be required for modulation of endogenous receptors. For example, even the highest doses of CNO used in the literature, 10 mg/kg^21^, would not lead to brain concentrations of above 70 nM and free (CSF) CNO levels above 30 nM, according to linear extrapolation of our data, which would still have minimal off-target effects.

In summary, our data highlight that CNO and Cmpd-21 would be suitable DREADD ligands and behavioural effects induced via DREADDs can be expected at latest from 15 min post injection onwards. Dose ranges of 3–5 mg/kg for CNO and 0.4–1.0 mg/kg for Cmpd-21 should be effective to activate hM4Di in mice *in vivo* and avoid unspecific behavioural effects. A dose range of 0.2–0.5 mg/kg for CLZ should be sufficient to activate the hM4Di in mice *in vivo* without inducing unspecific behavioural side effects. However, caution should be taken until any potential relative contribution of NorCLZ to the *in vivo* effects of CLZ in mice have been elucidated, to exclude NorCLZ as a confounding factor. In addition, proper between-subject controls are required to detect potential unspecific effects evoked by modulation of endogenous GPCRs by all three compounds.

## Methods

### Subjects

Thirty-two male C57BL/6 J mice (Charles River, Germany) were used in the behavioural experiments, which started at eight weeks of age. For the pharmacokinetic studies, eighteen 14–16-week-old C57BL/6 J mice (Charles River, Germany) were analysed. All mice were housed in groups of 2–4 in individually ventilated cages (IVC GM 500, Tecniplast, Germany) in a humidity- and temperature-controlled holding room under a 12 h light/dark cycle with food and water initially available ad libitum. Mice were permitted at least 5 days acclimatisation before handling and start of the behavioural study. Food restriction was initiated 3 days before the start of the behavioural training and was set to maintain the mice at 85–90% of their free-feeding weight.

All experimental procedures were authorised by the Local Animal Care and Use Committee (Regierungspräsidium Tübingen, approval number: 16–017-G) and were in accordance with local animal care guidelines and the Association for Assessment and Accreditation of Laboratory Animal Care (AAALAC) regulations, the German Animal Rights Law (Tierschutzgesetz 2013), and the EU Directive 2010/63/EU, as well as the United States Department of Agriculture Animal Welfare Act. All experimental studies were performed in an AAALAC certified facility and all experiments are reported in accordance with the Animal Research: Reporting of *In Vivo* Experiments Guidelines^25^.

### Measurement of efficacy at hM4Di

#### Primary neuronal culture

For the determination of the potency of CNO, CLZ, and Cmpd-21 at hM4Di *in vitro*, primary cortical neurons were prepared from brains of embryonic (E18) Sprague-Dawley rats (Janvier Labs, France)^19^. Briefly, cortices were dissected from embryonic brains, meninges were removed, and cortices transferred into ice-cold dissociation buffer (2 mM kynurenic acid, 10 mM HEPES, 20 mM MgCl_2_ (Fluka, Germany) and 33.4 mM Glucose in Hank’s Balanced Salt Solution (HBSS), pH 7.4 (Gibco, Thermo Fischer Scientific, Massachusetts, USA). Cortices were then dissociated in 20 units/ml papain solution (5.5 mM L- Cysteine hydrochloride, 1.1 mM ethylenediamine tetra-acetic acid [EDTA], 0.067 mM 2-Mercaptoethanol in Minimum Essential Medium [MEM]) for 15 min at 37 °C. Cortices were washed twice by adding 20 ml plating medium (1:50 foetal calve serum, 1:100 penicillin/streptomycin and 1:100 GlutaMax in MEM) followed by cell filtration using a 70 µm cell strainer (Corning, model #352350, Merck, Germany) to obtain a single-cell suspension. Dissociated cells were re-suspended in serum-free Neurobasal medium with Glutamax and B27 supplement (Gibco) and plated on PDL-coated 96 MTPs (Corning #356640). Virus (ssAAV-1/2-hSyn1-hM4D[Gi]_mCherry-WPRE-hGHp[A]) transduction was performed immediately after plating. Cells were maintained at 37 °C (5% CO_2_ and 10% O_2_) in a humidified incubator. A half feed medium change was done on DIV8. Final assay conditions used for evaluating activity of compounds were 0.95 × 10^5^ cells/cm^2^, transduced with 10.000 multiplicity of infection and measured on DIV9.

#### FLIPR assay

Medium was removed and cells were incubated in assay buffer (HBSS, Gibco, 10 mM HEPES, pH 7.4) containing 4 µM Fluo-4AM (Thermo Fischer Scientific) and 0.1% Pluronic F-127 (Thermo Fischer Scientific #P3000MP) for 1 h at room temperature in the dark. Loading buffer was then replaced with assay buffer. After 10 min, assay plates were measured in the FLIPR tetra (Molecular Devices, California, USA) using green fluorescence filter settings (excitation 470–495 nm, emission 515–575 nm). Fluorescence signals were recorded at 1 s intervals (0.4 s exposure time). Compound addition was automated in a single addition protocol. Tested compounds (concentrations:0.3 nM–1 µM; retigabine at 30 µM) were added after recording a baseline response for 5 min followed by another 5 min for recording compound effects on Ca^2+^ oscillations. Each compound dose was repeated on three individual plates in duplicates.

### Assessment of pharmacokinetic properties of CNO, CLZ, and Cmpd-21

The pharmacokinetic properties of 3.5 mg/kg CNO, 1 mg/kg CLZ and 3 mg/kg Cmpd-21 were assessed by collecting blood, CSF and brain samples at 15, 30 and 60 min after i.p. drug administration. Mostly, for each drug and time point six animals were used and analyzed, except for the 15 min time-point after CNO application (n = 5) and the CSF samples after CLZ injection where 5 samples were taken at 30 min and 7 samples at 60 min. Blood (approximately 100 µl) was collected from the *vena facialis* in anaesthetised animals, followed by CSF sampling (approximately 10 µl) from the *cisterna magna* under pentobarbital anaesthesia (1:25 in saline, 10 ml/kg, i.p., Narcoren, Boehringer Ingelheim, Germany). Subsequently, the brain was removed and its anterior third (including the medial prefrontal and anterior cingulate cortices) dissected and transferred into Precellys tubes cooled in liquid nitrogen. For plasma extraction, blood was transferred to EDTA coated tubes and centrifuged for 10 min at 10,000 rpm at 4 °C. Maximal plasma concentration (C_max_), time taken to reach maximal plasma concentration (T_max_) and mean residence time were assessed using liquid chromatography–mass spectrometry analysis executed on the HP1200 (Agilent, CA, USA) coupled to the API 6500 (AB Sciex, Germany) using BI00001052 (Boehringer Ingelheim) as internal standard. In short, 5 µl of plasma or CSF sample were mixed with 10 nl internal standard. 70 µl acetonitrile (ACN)/methanol (1:1) was added, kept at −20 °C for 15 min and centrifuged for 4 min at 4000 rpm at 4 °C. 30 µl of the supernatant was mixed with 170 µl 0.1% formic acid. Brain homogenization was executed by Precellys Evolution (Bertin) after adding ACN/methanol (1:1)/*aq*.*dest*. (3:1) to each brain sample with a volume corresponding to 4 parts of the brain weight, followed by centrifugation for 1 min at 4000 rpm at 4 °C. 5 µl of the supernatant was mixed with 10 nl of internal standard. A serial dilution was made by diluting the stock solution (2 mM, in dimethyl sulfoxide [DMSO]) of the compound to be tested, yielding concentrations of 0.5, 0.05, 0.005 and 0.0005 mM.

### Off-target screen

CNO and Cmpd-21 (at 10 µM, diluted in DMSO) were tested in binding and enzyme assays on 44 potential off-targets (Figs. 3 and 4). Receptors and transporters of interest were overexpressed in either human recombinant human embryonic kidney (HEK)-293 or Chinese hamster ovary (CHO) cells. Radio-labelled ligands (Figs. 3 and 4) were used to assess the binding ability of CNO and Cmpd-21 by scintillation counting. For ion channels, selective radio-labelled ligands (Figs. 3 and 4) were used and the binding ability of CNO and Cmpd-21 assessed using rat cerebral cortex tissue. Results are expressed as percent inhibition of control-specific binding of the radioactively labelled ligand specific for the respective target. For enzyme inhibition, the effect was calculated as a percent inhibition of control enzyme activity. Results showing an inhibition >50% were considered to represent significant effects of CNO or Cmpd-21. All presented values are the mean of two experiments.

For CNO, a dose-response follow-up study was conducted on the target receptors with > 50% inhibition of control specific binding (α1A, α2 A, dopamine D1, dopamine D2, histamine H1, muscarinic M1, M2 and M3, serotonergic 5-HT1A, 1B, 2B and 2A receptors). K_i_ was determined by calculating the values by non-linear regression of the competition curves generated with mean values of two replicates at eight different concentrations of CNO (0.3 nM–100 μM) using Hill equation curve fitting or using the Cheng-Prusoff equation.

### 5-Choice Serial Reaction Time Task

Training was performed in 16 standard mouse Bussey-Saksida touchscreen chambers (model #80614, Campden Instruments Ltd., UK), previously described in detail^26^. In short, each trapezoidal operant chamber was placed in a sound- and light- attenuating box individually equipped with a house light, a reward magazine with light, a liquid reward dispenser, a sound generator, a perforated stainless steel floor, and a touchscreen placed at the wide end of the chamber and covered permanently by a black acrylic glass 5-hole mask. The reward magazine, located opposite the touchscreen, was connected to a liquid dispenser delivering strawberry milk (Yazoo^®^). Infrared break-beam sensors located inside and on the front of the food magazine as well as on the touch-screen walls allowed the detection of reward delivery and the animal’s locomotor activity, respectively. Four individual chambers were connected to one computer for controlling the task flow using the graphical task design software ABET II Touch (model #89505, Campden Instruments Ltd., UK) and the WhiskerServer software (Cambridge University Technical Services Ltd., UK)^27^.

Mice were habituated to the chambers for three consecutive 20-min sessions during which the house light and magazine light were permanently on. At the start of each session, 30 µl strawberry milk was delivered. Every 15 s after head entry into the magazine, an additional 30 µl strawberry milk was delivered. Head entry turned the magazine light off.

The 5-CSRTT training protocol, originally designed for rats^28^, was similar as that previously described for mice^29–31^. The training comprised six stages with 30-min sessions initiated by the illumination of the house and magazine lights and delivery of 20 µl strawberry milk. Collection of the reward switched the magazine light off and started the first trial.

In the first stage, the middle window was permanently illuminated, which was turned off when touched, followed by reward delivery (20 µl) with illumination of the magazine light. Head entry switched off the magazine light. Mice performing 30 trials in a session continued to the next stage. Training sessions in stages 2–6 comprised a maximum of 120 trials. Collection of the reward initiated the inter-trial interval (ITI) followed by the illumination of one pseudo-randomly selected aperture for a fixed stimulus duration (SD). Any touch to the screen during the ITI was classified as a premature response which was not counted as a trial and terminated the current trial. Following stimulus detection, a nose poke to the corresponding aperture within a fixed time interval (limited hold; LH) was required for reward delivery. Premature responses made during the ITI, incorrect responses, and the omission of a response within the LH led to a timeout (TO, house light switched off for 4 s) instead of delivery of reward. Correct, incorrect and omitted responses defined the total number of trials. During stages 2–6, SD and LH were gradually decreased (SD: 32 s to 2 s, LH: 34 s to 4 s), whereas the ITI was increased once from 2 s to 5 s from stage 4 onwards. The criteria to pass the last stage and reach baseline performance were to complete >30 correct trials, with ≥80% accuracy and ≤30% omissions. Percentage accuracy was defined as the number of correct trials divided by the sum of correct and incorrect trials. Percentage omissions were calculated in terms of the number of completed trials. Premature responding was calculated as a percentage of completed trials. Perseveration was calculated as the number of additional responses made in the same aperture following a correct response. Additionally, the correct-response latency (time from stimulus presentation to correct response) and reward latency (time from correct response to reward collection) were measured across all trials.

To assess the effects of CNO, CLZ and Cmpd-21 on the cognitive domains of sustained attention and waiting impulsivity, mice performed 30-min challenge sessions with a reduced SD (0.8 s) and increased ITI (7 s). Either one or up to three days before, as well as one day after the task challenge, mice were trained at baseline conditions to check for any unexpected changes in task performance (e.g. by long lasting drug- or challenge-induced effects). Only mice reaching baseline performance before the challenge were included into the data analysis.

### Drugs

Drugs were administered according to a randomised latin-square design. CNO (Enzo Lifesciences, NY, USA) and CLZ (Sigma Aldrich, UK) were dissolved in hydrochloric acid (1:15) and 40% hydroxypropyl-β-cyclodextrine (1:10). The pH was adjusted to 6.5–7.5. Cmpd-21 (HelloBio, UK) was dissolved in 0.9% saline. The dose range and pre-treatment time were selected based on in-house pharmacokinetic data and published literature^32^. Since a fully automated data acquisition system was used, the experimenter was not blinded to treatment. All drugs were administered i.p. at a volume of 10 ml/kg 10 min before the start of the behavioural test. The applied doses were 3.5 or 10 mg/kg for CNO, 0.1, 0.3 or 1 mg/kg for CLZ, and 0.3, 1 or 3 mg/kg for Cmpd-21, in addition to vehicle in each case. Mice were habituated to injections by i.p. administration of 0.9% saline 3–5 days before drug administration.

### Data analysis

Time-series raw fluorescence traces recorded from the FLIPR assay were analysed by detecting Ca^2+^ peaks based on a fixed amplitude threshold^19^. The change in oscillation frequency was determined by counting the number of peaks in a defined time interval (5 min) before and after addition of the tested compound and calculating the Ca^2+^ oscillations frequency from their number. All data were normalised to baseline counts and then normalised to give a percentage response compared with vehicle control wells. For visualisation, all data were transferred to GraphPad Prism 7 (Graphpad Software, Inc., CA, USA).

Behavioural and pharmacokinetic data were analysed using GraphPad Prism 7. Pharmacokinetic data was collected in a between-subject design at multiple time points and analysed by one-way ANOVA and pairwise Tukey *post hoc* tests. Behavioural CNO data were analysed by pooling two independent experiments of which each had their own vehicle-controls and were conducted consecutively. Behavioural pharmacology data was analysed by repeated-measures ANOVA and Dunnett’s *post hoc* test in the case of within-subject designs with more than one dose of the agonist (CLZ, Cmpd-21 at the two lowest doses) or in the case of a within-subject design, where only one dose was tested against vehicle, a paired *t*-test was used (highest dose of Cmpd-21). Otherwise, an unpaired *t*-test was used (CNO). For repeated-measures ANOVAs, the Greenhouse-Geisser correction to the degrees of freedom and the *p*-values was applied, in case of a violation of sphericity indicated by Mauchly’s test.

Statistical significance was set at *p* < 0.05. Data are presented as mean ± standard error of the mean, individual data points are displayed wherever applicable.

## Data Availability

All data generated and analysed during this study are included in this article. Numeric source data for the presented figures are available from the corresponding authors on reasonable request at the desired level of analysis.
